# Competition among *Escherichia coli* Strains for Space and Resources

**DOI:** 10.3390/vetsci5040093

**Published:** 2018-11-02

**Authors:** Sarah-Jo Paquette, Rahat Zaheer, Kim Stanford, James Thomas, Tim Reuter

**Affiliations:** 1 Alberta Agriculture and Forestry, #100-5401 1st Ave. S, Lethbridge, AB T1J 4V6, Canada; sarahjo.paquette@uleth.ca (S.-J.P.); kim.stanford@gov.ab.ca (K.S.); 2 Department of Biological Sciences, University of Lethbridge, 4401 University Drive W, Lethbridge, AB T1K 3M4, Canada; thomas@uleth.ca; 3 Agriculture and AgriFood Canada, #205-5403 1st Ave. S, Lethbridge, AB T1J 4P4, Canada; rahat.zaheer@canada.ca

**Keywords:** *E. coli*, Shiga toxin producing *E. coli*, competition, probiotics, interactions, warfare

## Abstract

Shiga toxin-producing *Escherichia coli* (STEC) are a subgroup of *E. coli* causing human diseases. Methods to control STEC in livestock and humans are limited. These and other emerging pathogens are a global concern and novel mitigation strategies are required. Habitats populated by bacteria are subjected to competition pressures due to limited space and resources but they use various strategies to compete in natural environments. Our objective was to evaluate non-pathogenic *E. coli* strains isolated from cattle feces for their ability to out-compete STEC. Competitive fitness of non-pathogenic *E. coli* against STEC were assessed in competitions using liquid, agar, and nutrient limiting assays. Winners were determined by enumeration using O-serogroup specific quantitative PCR or a semi-quantitative grading. Initial liquid competitions identified two strong non-pathogenic competitors (O103F and O26E) capable of eliminating various STEC including O157 and O111. The strain O103F was dominant across permeable physical barriers for all tested *E. coli* and STEC strains indicating the diffusion of antimicrobial molecules. In direct contact and even with temporal disadvantages, O103F out-competed STEC O157E. The results suggest that O103F or the diffusible molecule(s) it produces have a potential to be used as an alternative STEC mitigation strategy, either in medicine or the food industry.

## 1. Introduction

In 1934 the Russian biologist, Georgii Gause conceived that rivals for limiting resources cannot coexist and that one competitor would become “prey” and the other “predator”, demonstrating this concept using yeast and protozoa [1]. This became known as the “Competitive Exclusion Principle” [2], which states “Complete competitors cannot coexist”. Accordingly, in an environment where one species has an advantage such as increased growth rate, the species with the advantage (predator) will dominate over the long term and eliminate the weaker competitor (prey).

**Figure 1:**
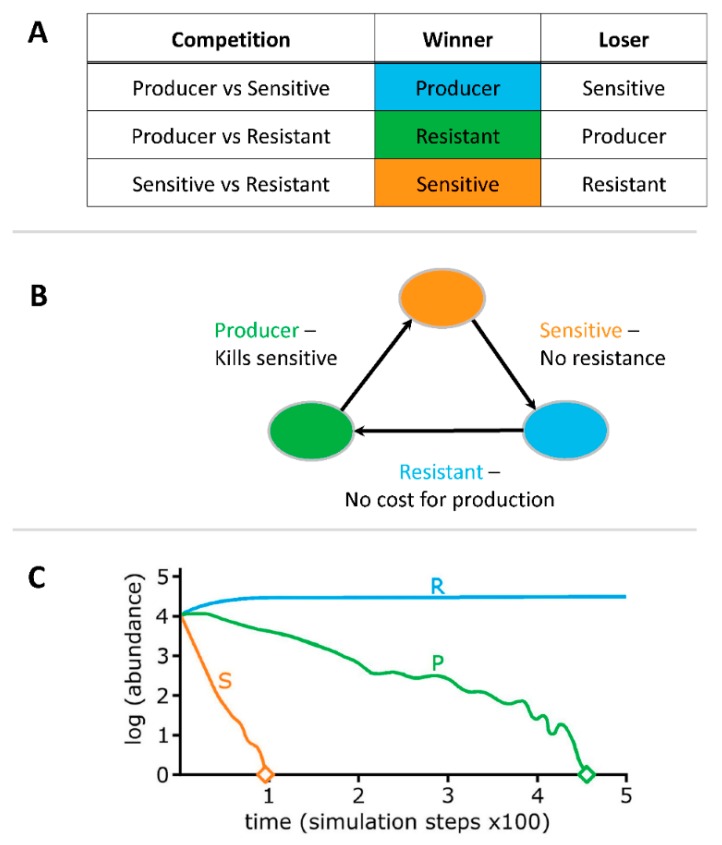
“Rock-Paper-Scissor” game of competition dynamics. Note: R = resistant, P = producer and S = Sensitive. Compartments (**A**), (**B**) and (**C**) are adapted from references [6,7,8], respectively.

Environments populated by *Escherichia coli* are subject to competition pressures due to limited availability of space and resources. Bacteria use various weapons to ensure their survival [3] and ability to compete which can take place in any natural environment [4,5]. Competition can be exploitative where a predator restricts nutrients and starves the prey [4]. Effective exploiters can prevail in competitions, but weak exploiters can also out-compete a vigorous exploiter using interference competition. Interference-based competition uses antagonistic factors produced by competitors such as toxins to prevent or remove competitors from their environment [4]. The competitive outcome depends on the dynamic of the competitive interactions, which is often referred as the “rock-paper-scissors” game (Figure 1) [6].

Producers generate molecules such as antibiotics, specialized enzymes, and colicins that can kill, prevent growth and/or disrupt signal cascades [4]. However, there is a high cost to producing molecules such as colicins and in a two-strain system, removal of the sensitive strain provides an advantage to the producer (Figure 1A,B) [8]. In contrast, sensitive strains do not have any metabolic cost for either producing or resisting, providing a metabolic advantage in competitions with resistant strains, while succumbing in the presence of producers. Resistant strains, avoid metabolic costs associated with molecule production, but there are costs associated with resistance, which are less than production but higher compared to sensitive strains. In a three-strain dynamic with no spatial structure (well-mixed conditions, such as an aquatic environment) competitive advantage shifts to resistant strains (Figure 1C) [8,9].

Interference based competition can be divided into independent and contact-dependent methods [10]. Contact independent mechanisms rely on diffusible compounds such as bacteriocins and antibiotics to damage and/or kill competing bacteria or signals such as quorum-sensing molecules to facilitate interactions between bacteria. Direct-contact interactions also occur with contact-dependent growth inhibition (CDI), which requires specific receptors on competing cells or type VI secretion systems (T6SS), which do not need specific targets [10,11]. *E. coli* has been shown to possess all three types of interference-based competitive mechanisms [11,12,13].

*Escherichia coli* is a commensal bacterium and part of the gastrointestinal microbiota in humans and livestock. While many *E. coli* are considered harmless or beneficial, others such as Shiga-toxin producing *E. coli* (STEC) are virulent causing gastrointestinal diseases [14]. STEC are classified by the ability to produce at least one Shiga toxin (Stx) [15] and can cause severe infections such as hemolytic uremic syndrome or hemorrhagic colitis [16,17].

Ruminants, especially cattle, are a major host of STEC and many technologies to control STEC in livestock have been evaluated including vaccines, direct-fed microbials, and tannins [18,19,20]. Ultimately, none of these has shown consistent efficacy. As well, treatment options for humans infected with STEC are limited as some antibiotics may increase Shiga toxin production and/or release by inducing the bacterial SOS response [21]. As dependable methods to control STEC in cattle and humans are not yet available, use of non-pathogenic *E. coli* predator strains to out-compete and control pathogenic STEC strains may have potential. Almost 100 years ago, *E. coli* strain Nissle was recovered from the gut of a soldier and has been subsequently used as a probiotic to mitigate intestinal infections in humans [22]. Similarly, another study reported *E. coli* O157:H7 mitigation in cattle after the use of a direct-fed microbial consisting of non-pathogenic *E. coli* strains [23].

Our primary objectives were to investigate competitive non-pathogenic *E. coli* strains isolated from cattle feces with the potential to control STEC such as O157:H7 and investigate the competitive mechanisms utilized by these predator strains and potentially identify if these strains were producers and/or resistant.

## 2. Materials and Methods

### 2.1. Bacterial Strains: Cultures, Media and Culture Conditions

All strains used in this study were isolated from feces collected from transport trailers of slaughter cattle at two Alberta slaughter plants [24] (Table 1). *E. coli* were streaked from glycerol stocks onto MacConkey Agar (MAC, BD, Sparks, NV, USA). Plates were incubated overnight (16–18 h) at 37 °C. A single colony was selected from each plate and inoculated into 10 mL *E. coli* broth (EC) (EMD Millipore, Etobicoke, ON, Canada) and incubated overnight at 37 °C statically (liquid competition) or with shaking at 150 rpm (other competitions).

### 2.2. Competition Experiments

Various *E. coli* strains were examined for competitive fitness using tests to identify strong candidates that prevented or modified growth of STEC.

#### 2.2.1. Liquid Competition

The liquid competition was adapted from that previously described [25]. Overnight cultures of each strain were diluted to a starting cell density of ~1000 cells based on optical density (OD) measured at a wavelength of 600 nm. Both competitors (100 µL each) were added to EC for a final starting volume of 10 mL and grown 24 h at 37 °C, statically. A pure culture control (100 µL in EC) of each competitor was also prepared and grown under the same conditions as the competition cultures. After 24 h, 100 µL from each competition and control were inoculated into 9.9 mL of fresh EC and grown for another 24 h, with this repeated for a total of 14 days. Samples were removed (100 µL of culture) at time = 0, 3, 7, and 14 days for quantitative real-time PCR (qPCR) monitoring of copy numbers of O-serogroup specific gene sequence to extrapolate cell density of a particular strain in the culture. Primers, probes, and PCR conditions were performed, as previously described [25].

#### 2.2.2. Omelette Method

**Figure 2:**
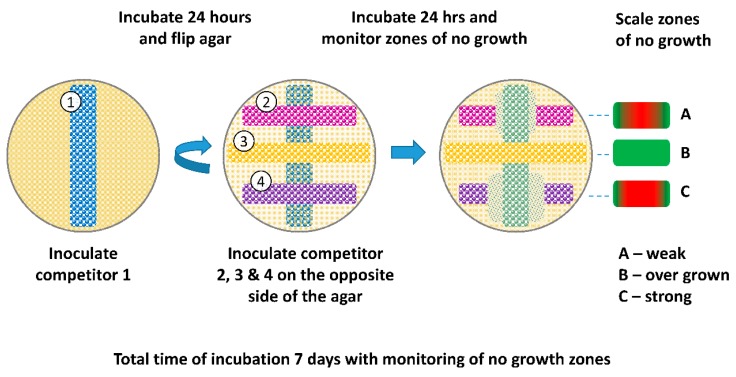
Schematic of Omelette method. Competitor strains were streaked on both surfaces of a 4 mm MAC plate. The bottom strain was streaked and grown for 24 h at 37 °C. After 24 h, the agar was flipped and three strains were streaked perpendicular to the bottom strain, separated by the thickness of the agar. Plates were then incubated for an additional 24 h and examined for zones of no growth graded by a scale of 1-to-10. Plates were incubated for another 6 days (7 days total) to monitor changes in zones of no growth. Agar thickness of 4, 7, and 10 mm were evaluated.

After overnight incubation, one competitor was streaked across a 4 mm MAC plate using a cotton swab. The hereinafter bottom strain was then incubated for 24 h at 37 °C. After 24 h, the agar was flipped and 3 strains were streaked perpendicular to the bottom strain and separated from the bottom strain by the thickness of the agar (Figure 2). Plates were then incubated for an additional 24 h at 37 °C and examined for zones of no growth directly over the bottom strain and graded using a scale of 1-to-10, with 1 being no growth over the bottom strain and 10 being full growth. Plates were incubated for an additional 6 days (7 days total) at 37 °C to monitor changes in zones of growth inhibition. A second trial was performed, as described above, with the following change: Agar thickness of MAC plates was increased to 7 mm and 10 mm and compared to the original 4 mm thickness.

#### 2.2.3. Plug ‘n’ Prey

**Figure 3:**
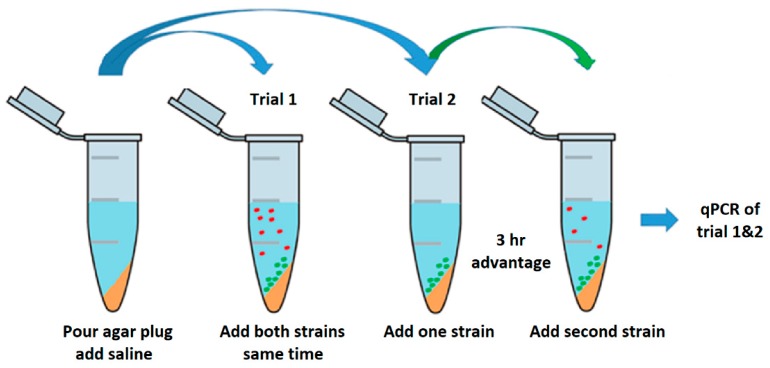
Schematic of Plug ‘n’ Prey. A 2 mL tube prepared with slants of 200 µL MAC agar. Slants were overlaid with 800 µL saline buffer (0.9%). In the first trial, overnight cultures were diluted to an OD 600 nm of 0.100 and grown to an OD 600 nm of 0.3–0.4 to ensure actively growing cells. Actively growing cells were diluted to a starting concentration of ~1000 cells and 100 µL of each competitor was inoculated at same time. The second trial was performed, as described, with the following change: One strain was inoculated 3 h prior to the competitor. Samples for both trials were taken at time points 0, 2, 4, and 6 days. Tube contents were divided into supernatant and slant and individually analyzed by qPCR monitoring copy numbers of O-serogroup specific gene fragment amplification.

The competition assay was performed in 2 mL tubes prepared with slants of 200 µL MAC agar. Slants were overlaid with 800 µL saline buffer (0.9% NaCl). Overnight cultures for both trials were prepared as previously described and were diluted to an OD 600 nm of 0.100 and grown to an OD 600 nm of 0.3–0.4 at exponential cell growth. Actively growing cells were diluted to a total starting cell density of ~1000 cells. In the first trial 100 µL of each competitor was inoculated into the 2 mL tubes at the same time (Figure 3). Samples were taken at time points 0, 2, 4, and 6 days. Tube contents were divided into supernatant and slant and individually analyzed by qPCR targeting O-serogroup specific gene to monitor the copy numbers. The second trial set-up was equivalent to the first except one strain was inoculated into the 2 mL tube 3 h prior to the competitor.

### 2.3. Statistical Analysis

Numerical data generated by qPCR for the liquid competition and plug ‘n’ prey were examined for normal distribution and the data were log transformed prior to analyses. Serogroup, competition, control, and interactions were determined for both liquid and plug ‘n’ prey using a mixed linear model (Proc Mixed, SAS 9.4, SAS Institute Inc., Cary, NC, USA). Given *p* values < 0.05 were considered significant.

## 3. Results

### 3.1. Liquid Competition

**Figure 4:**
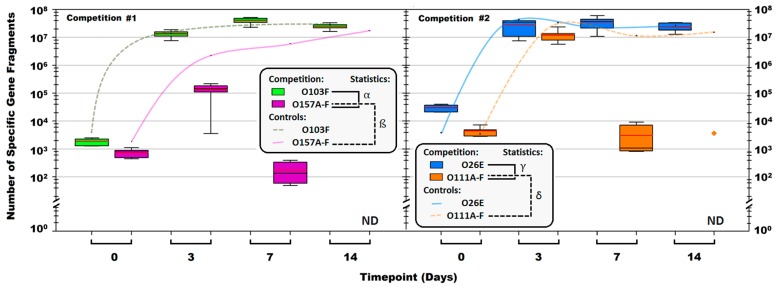
Total number of *Escherichia coli* O103F, O157A-F, O26E and O111A-F specific gene fragments in each liquid competition and corresponding pure culture controls calculated using qPCR. The boxes are serogroup specific enumerations for competition cultures and the lines are the pure culture controls. Note: ND = not detected. Red line in the boxes is the average proportion for the group and black line in the boxes is the median. The 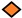 symbol denotes the O111B strain that was still present after 14 days. Symbols: α, β, γ, and δ denote a significant difference between: O103F and O157A-F in competition, O157A-F in competition and O157A-F controls, O26E and O111A-F in competition and O111A-F in competition and O111A-F controls, respectively (*p* < 0.05).

The qPCR enumerations from 72 competitions of either O103 (six non-pathogenic strains tested) vs. O157 (six STEC tested) or O26 (five non-pathogenic strains and one STEC) vs. O111 (six STEC) identified strong non-pathogenic *E. coli* competitors that were able to eliminate O157 and O111 (Figure 4). The O103 vs. O157 competitions identified a strong strain, O103F that eliminated all six opponent strains of O157 tested to levels undetectable by qPCR by day 14. Similarly, a strong non-pathogenic O26, O26E, eliminated five of six pathogenic O111 with only O111B remaining after 14 days and present in lower numbers (10^3^) compared to O26E (10^7^). Growth of 103F was greater than each O157 tested and growth of O26E exceeded that of each O111 (*p* < 0.05). Growth of pure culture controls for each competition set O103F, O157A-F, and O26E, O111A-F did not differ (*p* > 0.05) and all were present over the 14 days (Figure 4).

### 3.2. Omelette Method

The semi-quantitative data from the first trial (4 mm MAC plates) with various strains also identified the O103F from the liquid competition as a strong competitor (Supplemental, Figure S1). O103F had a strong zone of no growth at 24 h and maintained the zone for seven days, while the O157 initially had a less pronounced zone of no growth at 24 h and at day seven the strong O103F was grown over the original no growth zone of O157A. From the 4 mm thickness trial, another strong competitor was identified, *E. coli* O178A, which maintained a strong zone of growth inhibition over seven days against the O103F and O26E strains from the liquid competition (Supplemental, Figure S2a). Furthermore, both the O103F and O178A strains maintained stronger zones of no growth compared to O26E, which had a less prominent zone of no growth (Figure S2a,b).

**Figure 5:**
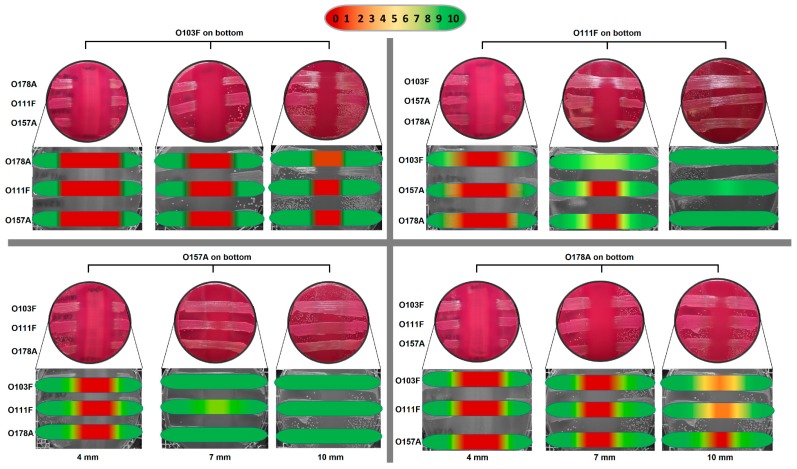
Omelette method results for *Escherichia coli* O103F, O111F, O157A, and O178A with varying plate thickness (4, 7, and 10 mm) against each other examining zones of no growth. Growth zones are graded beneath each plate with red (0) representing no growth to green (10) representing no inhibition of growth.

The 24 h semi-quantitative data from the second trial (four, seven, and 10 mm MAC plates) with O103F, O178A, O111F, and O157A demonstrated that at four mm agar thickness, all four strains had a strong zone of no growth and as agar thickness increased the size of the zones of growth inhibition varied by strain (Figure 5). O157A did not maintain a zone of no growth at seven mm and all three competing strains (O103F, O178A, and O111F) had solid growth when O157A was the bottom competitor. In comparison, O111F at 7 mm maintained the zone of no growth only for O157A and O178A, but not O103F. Both O178A and O103F maintained a narrowed zone of no growth at seven mm but at 10 mm thickness, O103F still maintained a zone of no growth for O111F and O157A and very limited growth for O178A. In contrast, O178A at 10 mm maintained the zone of no growth only for O157A with both O111F and O103F growing over the bottom O178A strain.

### 3.3. Plug ‘n’ Prey

**Figure 6:**
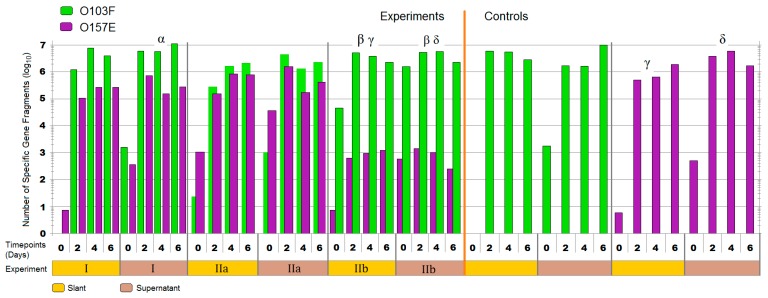
The total number of *Escherichia coli* O103F and O157E specific gene fragments in the supernatant and on the agar slant, across three plug ‘n’ prey experiments in comparison to the controls at each time point. Note: **I** = O103F and O157E added at the same time (bars side by side), **IIa** = O157E 3 h advantage over O103F (O157E bar on top), **IIb** = O103F 3 h advantage over O157E (O103F bar on top). Timepoint 0 is first time point after addition of both competitors. Symbols: α, β, γ, and δ denote a significant difference between: O103F and O157E in experiment **I**—supernatant, O103F and O157E in experiment **IIb**—slant and supernatant, O157E in experiment **IIb** and O157E control—slant and O157E in experiment **IIb** and O157E control—supernatant, respectively (<0.05).

The qPCR enumerations for plug ‘n’ prey demonstrated the ability of O103F to out-compete O157E when nutrients were limited (Figure 6). The first trial (**I**) with O103F and O157E in competition demonstrated that by day six, O103F was 10 times more numerous in both matrices, slant and supernatant, but differences in growth between O103F an O157E were found only for the supernatant (*p* < 0.05). The second experiment (**IIa**) with O157E having a three h advantage demonstrated that O103F overcame the disadvantage by day two and had 10 times higher concentrations of cells by day six, although overall growth did not differ (*p* > 0.05) between O103F and O157E. When O103F had the three h advantage (trial **IIb**) it won by 30 times higher concentrations in comparison to the O157E for the slant environment and was 40 times higher in the supernatant (*p* < 0.05). Comparing the O157 pure culture controls to O157 across all three competitions showed higher growth (*p* < 0.05) controls of O157E, as compared to competition for trial **IIb**. In contrast, even in competition, O103F grew similar to the O103F control in all trials (*p* > 0.05).

## 4. Discussion

In three different settings, the competition of *E. coli* strains for resources and/or space was monitored. Using liquid media permitted cell-to-cell contact, while solid media created a physical barrier between opposing strains. Previous research reported that interference-based competitions can be contact-dependent or contact independent [10], although when using a physical barrier only a producing predator would win competitions for space. During liquid competitions, both distant and close combat tactics were applicable. However, within the homogeneous distribution of cells and nutrients in liquid, it cannot be determined if coordinated strategies by strains took place or if one competitor conquered due to an advantage in metabolism and/or proliferation.

### 4.1. Liquid Competition

Using liquid competition, we identified two non-pathogenic *E. coli* strains (belonging to serogroups O103 and O26), which significantly reduced opponent strains to below limits of detection or resulted in a 4-log_10_ reduction of six different strains each of O157 (vs. O103F) and O111 (vs. O26E) (*p* < 0.05). This finding, that non-pathogenic *E. coli* have the ability to outcompete pathogenic *E. coli* strains, is in accordance with a previous study reporting that calves treated with probiotic *E. coli* shed significantly less O26:H11 and O111:NM STEC compared to untreated calves [26]. Furthermore, strong competitors within our *E. coli* strains were previously identified [25]. However, O26C, a strong competitor previously reported [25] was weaker compared to O26E identified in this study.

Competition can be exploitative or interference based [4] and it is possible that the O103F and O26E identified in the liquid competition are great exploiters and simply out-competed their competitors for resources. However, the qPCR enumerations for all the pure-culture controls suggest otherwise since all strains proliferated to similar concentrations in EC without competition (*p* > 0.05), suggesting that the liquid competition results are due to interference and not exploitation.

*E. coli* is known to have both contact independent and dependent interference-based systems and have been shown to produce various bacteriocins known as colicins [12], may possess a CDI system [11] and/or harbor a T6SS system [13]. In a pure culture of planktonic bacteriocin producing *E. coli*, 0.5–3% of the population express bacteriocin spontaneously [27]. Possibly, O103F and O26E expressed bacteriocins out-compete the O157 and O111 strains tested. CDI systems in *E. coli* have also been shown to be active in liquid culture as *E. coli* EC93 inhibited *E. coli* K-12 cells [28]. Moreover, CDI systems require specific receptors on the competing bacterial cells that are often found on closely related strains or within the same species, suggesting the CDI mechanism could be employed during competition. Contrary to CDI systems, the T6SS targets cells non-specifically by using physical forces to deliver effectors [10]. Here, T6SS was an unlikely mode of action, since a highly-active T6SS competitor was unable to target sensitive strains in liquid medium in contrast to a solid medium [29], which suggests that, in liquid competitions, bacteria do not use T6SS, but rely on other mechanisms.

### 4.2. Omelette Method

The results from the semi-quantitative omelette assay revealed the production of diffusible toxins or noxious products since the competitor strains were separated by a physical barrier preventing contact-dependent competition. Agar plates are often used to visualize competitive interactions due to the manifestation of visible phenotypes that identify competition winners [9]. Similar to the results of the liquid competition, O103F produced a stronger diffusible substance than the competitors. Here, we also identified another strong contact–independent strain, (O178A) with zones of no growth similar in size to those of O103F. In comparison, the O26E identified in the liquid competition did not have as strong of a zone of no growth as O103F, suggesting the presence of a weaker diffusible substance. The second trial further demonstrated the strength of the O103F diffusible substance through distance. O103F maintained the strongest zone of no growth as agar thickness increased for all strains tested including O178A from the first trial. Overall, while O103F appeared to have the strongest diffusible substance and best resistance, all strains tested showed an initial zone of no growth at 24 h, suggesting that they all produced a detrimental substance capable of preventing growth of other *E. coli*, which conforms to previous reports that *E. coli* produces diffusible toxins [12,30]. Furthermore, various studies have examined *E. coli* strains for production of bacteriocins and the percentage of strains that produce bacteriocins can vary from 10% to 70% depending on the environment where they were isolated [31].

Bet-hedging is a survival tactic used by bacteria, where they express phenotypes randomly instead of in reaction to environmental cues [32]. Bacteriocin production has been shown to occur at a low frequency in growing producer populations [27] and a recent study has shown that *E. coli* colicin producers use bet-hedging as a survival strategy [32]. A previous study suggested that such a low-level production could be considered a pre-emptive attack against sensitive strains [33]. Possibly, the diffusible molecule produced by O103F and others was spontaneously produced during the first 24 h of the omelette study providing an advantage to the bottom strain prior to addition of the competitors. Furthermore, low-level producers in an established colony can signal sister cells to mount a collective attack against invading cells [33]. Potentially, if some O103F cells in the established colony were already producing bacteriocins, these cells could have sensed the competitors and signaled sister O103F cells, mounting stronger attacks against the “invading cells” indicated by wider zones of no growth. On the other hand, bacteriocins have been suggested to act first as a signaling and repelling molecule rather than being lethal and possibly the competitors of O103F, which were repelled by the molecule produced by O103F [34].

Based on the classification of competitors in a bacterial warfare as either producers, resistant or sensitive [6] (Figure 1), all strains tested in this study appear to be producers. However, revealing the characteristics of a producer, strain O103F can also grow over other strains over time, implying that O103F is both a strong producer and a resistant strain. Bacteriocin production is based on the translation of three genes encoding toxin, immunity, and lysis [32]. Therefore, production confers immunity and likely any diffusible molecule will be paired with the production of immunity molecules to prevent the killing of sister cells. *E. coli* strains have been shown to produce more than one colicin and microcin [30] and possibly O103F is resistant to some bacteriocins and/or diffusible(s) from other *E. coli* strains because O103F produces a similar diffusible, which confers immunity. On the other hand, immunity can also be conferred by mutations that either alter receptors or the translocation system for the bacteriocin [6,30] and O103F may be resistant to diffusible(s) of other *E. coli* strains due to these types of mutations. Future research on the specific O103F diffusible(s) may determine if (**I**) the diffusible is a bacteriocin, (**II**) the diffusible is killing or signaling competitors and (**III**) the diffusible immunity is conferred by production of the diffusible or due to mutations that grant immunity, which may further elucidate the strength of the O103F strain as a competitor.

### 4.3. Plug ‘n’ Prey

This study was designed to examine the effect of limiting nutrients on competition outcome between the identified strong O103F and the previously tested O157E strain. Limiting nutrients did not change the overall outcome in all the trials and O103F won all the competitions, including when inoculated with a three h disadvantage into vials containing the nutrients but growth between O103F and O157E was only different (*p* < 0.05) for trial **I**—supernatant and trial **IIb**—both matrices. Perhaps, the lack of nutrients in plug ‘n’ prey affected the ability of O103F to compete effectively once nutrients were depleted. Previous research has shown that bacteriocin production in a lactic acid bacterium was modified when the carbon source changed and bacteriocin production increased or decreased depending on the carbon source [35]. Conceivably, the same modification of bacteriocin production may be seen by limiting the carbon source.

Ultimately, O103F won both competitions having either an advantaged or disadvantaged access to limited nutrients. It overcame the O157E advantage to win overall and prevented O157E from growing past the inoculation density of 1000 cells when O103F had the advantage. Having the advantage, O103F won all competitions, which is in accordance with a previous study that reported that an “established” colony is more successful mounting attacks against invading competitors [33]. With a three h advantage, O103F suppressed the growth of O157E over six days by preventing O157E from growing beyond 1000 cells. However, the “established” O157E was not able to prevent O103F from growing, which is contrary to previous reports [33]. In the end, after a three h advantage O103F was able to maintain a competitive advantage, while O157E was not able to fortify the nutrient source during this time, further demonstrating the predatory strength of O103F compared to O157E. Future studies with different nutrients and different advantage times may further elucidate if and how the competitive mechanisms of O103F are affected by nutrition.

Bacteria, such as *E. coli* are found in almost every habitat on earth and face fierce competition for space and resources [5,10,36]. In order to ensure their survival, bacteria use various competitive mechanisms which can be exploitative and/or interference based [4,10]. Among others, habitats for virulent *E. coli* are humans and their food sources. During medical treatment and along the food production chain, control of proliferation, and/or colonization by virulent *E. coli* remains a challenge.

STEC are a significant food borne pathogen [37] and are classified by the ability to produce at least one Stx [15]. Cattle are considered the main reservoir for STEC and STEC carriage in cattle is asymptomatic due to a lack of receptors for Stx [38]. On the other hand, Stx is considered a main virulence trait to cause human disease [39] since Stx binds to globotriaosylceramide (Gb3) present on endothelial cells [37]. Benefiting from natural competitiveness, *E. coli* champions may offer mechanisms to mitigate STEC as protective culture and/or additive within the food chain or as a probiotic treatment option for human infections.

It should be noted that highly-competitive non-pathogenic *E. coli* may have the potential to become STEC if infected with a Stx bacteriophage. However, a study examining the ability of various stx2-phages to infect *E. coli* from different pathotypes found that while all strains could be infected with Stx-phages, not all Stx-phages infected every *E. coli* and phage integration was rarely stable [40]. Other studies examining Stx-phage infection on various food sources found that for Stx-phage infection to occur both the donor and recipient need to be present in high concentrations not typically found in food samples [41,42]. Together, these studies suggest that while Stx-phage infection is possible: (1) It is unlikely to occur and (2) rarely is phage integration stable. Unless there is stable integration, the phage DNA is removed, rendering *E. coli* a non-STEC. Furthermore, identification of the diffusible(s) produced by these highly competitive non-pathogenic *E. coli* would mitigate the risk by removing the need to use the bacteria and instead only use their products.

This study identified a strong O103 competitor based on three different experimental settings against various strains including STEC O111 and STEC O157. The exact mode of action used by O103F to out-compete other *E. coli* strains remains unknown but O103F likely produces at least one diffusible substance that affects the viability of other *E. coli*. Diffusible molecules produced by *E. coli* can be colicins, antibiotics, or quorum sensing molecules [4], and are potential alternatives to antibiotics [43]. Future evaluation of *E. coli* O103F may identify the effective diffusible substance(s) produced by this strain and may provide an alternative STEC mitigation strategy as therapeutic treatment or protective culture in the food industry.

## 5. Conclusions

Among living organisms, fierce battles exist to secure habitats and natural resources or even for survival. Between bacterial competitors, predators, and prey have developed several strategies to protect their existence and survival combat interactions that might ultimately be correlated to energy conversion efficiencies and the capacity to proliferate. Our observations revealed a number of highly competitive *E. coli* strains, but ended with one exclusive champion. Over 100 years ago, a champion (*E. coli* Nissle) from a human host was discovered to battle virulent bacteria and has been successfully marketed as a probiotic, mitigating human infections since then. Novel emerging pathogens are a global concern and new approaches for mitigation strategies require further evaluation. Here, numerous Shiga-toxin producing *E. coli* strains were outcompeted by a non-pathogenic *E. coli* strain that was isolated from cattle feces. This non-pathogenic strain shows the potential to be used to control pathogenic *E. coli* that compromise health and/or food safety.

## Figures and Tables

**Table 1:** *Escherichia coli* strains utilized in this study.

Rank	Serogroup		H-Type	Toxin	eae	hlyA	Liquid	Omelette	Plug n’ Prey	Discussed in Manuscript
**Strongest**	O103F		NM	−	−	−	√	√	√	√
**Strong**	O26E		H9	−	−	−	√	√		√
	O178A		H7	−	NT	NT		√		√
	O178B		NT	−	−	−		√		
**Weak**	O26	A	NT	−	−	NT	√			
		B	NT	−	+	NT	√			
		C	H18	−	−	−	√			
		D	H11	*stx 1*	+	+	√	√		
		F	NM	−	+	−	√			
	O45	A	H4	−	−	−		√		
		B	NT	*stx 1*	+	NT		√		
		C	NT	−	−	NT		√		
	O51A		NM	−	−	−		√		
	O103	A	NM	−	+	+	√			
		B	NM	−	+	+	√			
		C	NM	−	+	+	√			
		D	H38	−	−	−	√			
		E	NM	−	+	+	√	√		
	O111	A	NM	*stx 1*	+	+	√	√		
		B	NM	*stx 1*	+	+	√			
		C	H8	*stx 1*	+	+	√			
		D	H8	*stx 1*	+	+	√			
		E	NM	*stx 1*	+	+	√			
		F	NM	*stx 1*	+	+	√	√		√
	O145	A	NM	*stx 1*	+	+		√		
		B	H25	−	+	+		√		
	O157	A	H7	*stx 1 & 2*	+	+	√	√		√
		B	NT	*stx 1 & 2*	+	NT	√			
		C	NT	*stx 1 & 2*	+	NT	√			
		D	H7	*stx 1 & 2*	+	+	√			
		E	NT	*stx 2*	+	NT	√	√	√	√
		F	NT	*stx 1 & 2*	+	NT	√			

Note: NT = Not tested, Symbol: − = tested and not present, + = tested and present. Multiple representative strains of each serotype were used in this study. (√) checkmark symbol identifies which strains were used in each test and which strains are discussed in the manuscript. STEC (Shiga toxin-producing Escherichia coli) were defined as Escherichia coli strains positive for at least one shiga toxin gene by PCR.

## Supplementary Materials

The following are available online at http://www.mdpi.com/2306-7381/5/4/93/s1, Figure S1. Omelette method results for O103 and O157 at 24 h and 7 days against each other examining zones of clearing, Figure S2. Omelette method results for O26, O103 and O178 against each other examining zones of clearing at day 7.
