# Supplementary material for: Competition among Escherichia coli Strains for Space and Resources
Source: Vet Sci. 2018 Nov 2;5(4):93. doi: 10.3390/vetsci5040093 (PMC6313926; doi:10.3390/vetsci5040093)
Supplement: Supplementary file 1 [file vetsci-05-00093-s001.pdf]

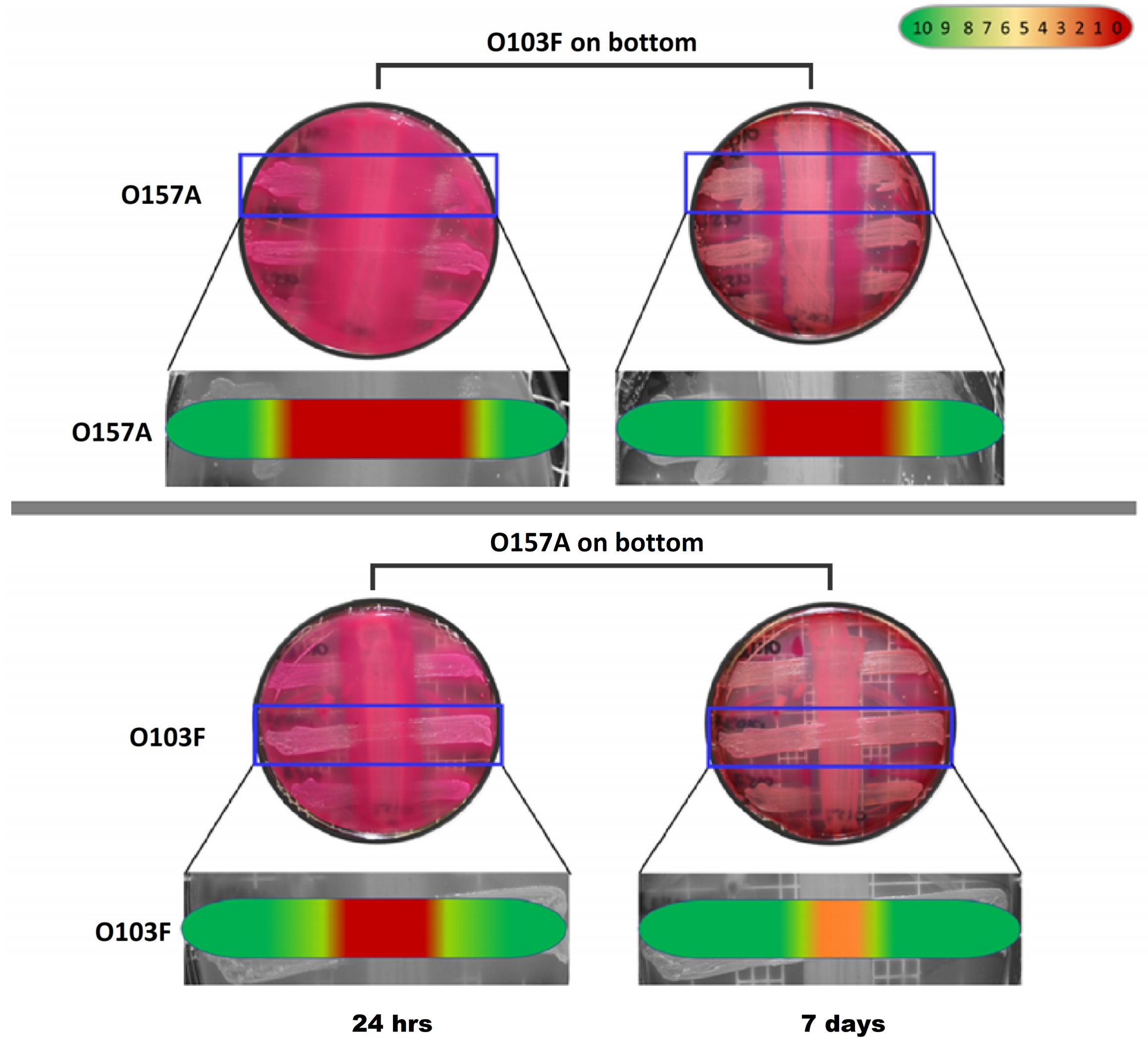

Figure S1. Omelette method results for O103F and O157A at 24 hrs and 7 days against each other examining zones of clearing. Zone of clearing strength is graded with green (10) being thick growth and red (0) being no growth on plates. Note: Selection corresponds to either O103F or O157A, other weak competitors were also grown on the plates but they are not discussed within the paper.

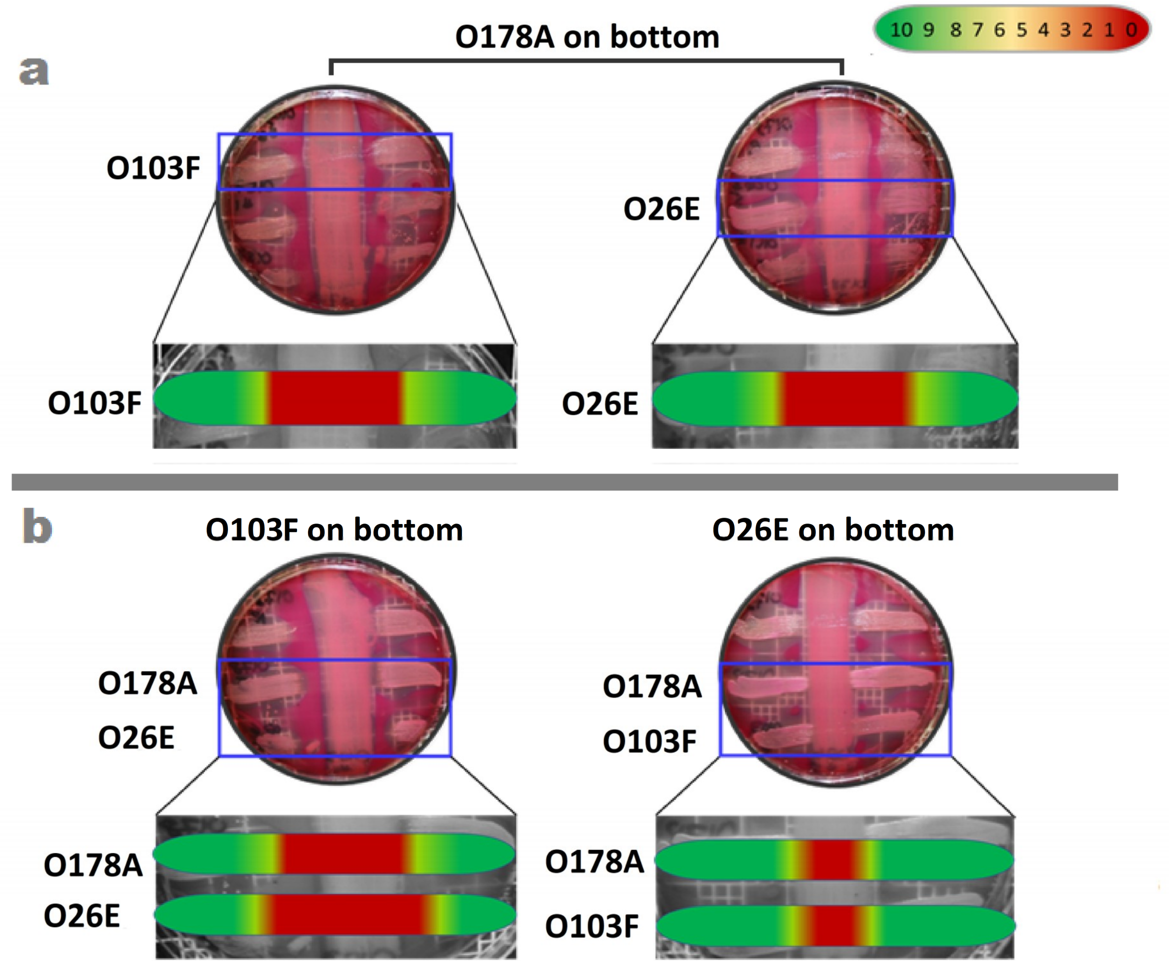

Figure S2. Omelette method results for O26E, O103F and O178A against each other examining zones of clearing at day 7. Zone of clearing strength is graded with green (10) being thick growth and red (0) being no growth on plates. Note: Selection corresponds to O26E, O103F or O178A, other weak competitors were also grown on the plates but they are not discussed within the paper.
